# The effects of smoking and smoking cessation on nasal mucociliary clearance, mucus properties and inflammation

**DOI:** 10.6061/clinics/2016(06)10

**Published:** 2016-06

**Authors:** Daniela Mitiyo Odagiri Utiyama, Carolina Tieko Yoshida, Danielle Miyuki Goto, Tômas de Santana Carvalho, Ubiratan de Paula Santos, Andreas Rembert Koczulla, Paulo Hilário Nascimento Saldiva, Naomi Kondo Nakagawa

**Affiliations:** I Faculdade de Medicina da Universidade de São Paulo, Departamento de Fisioterapia, Fonoaudiologia e Terapia Ocupacional</org-name>LIM-34, São Paulo/SP, Brazil; II Faculdade de Medicina da Universidade de São Paulo, Departamento de Patologia, LIM-5, São Paulo/SP, Brazil; III Universidade Nove de Julho, Departamento de Fisioterapia, São Paulo/SP, Brazil; IV Faculdade de Medicina da Universidade de São Paulo, Heart Institute (InCor), Pulmonary Division, Smoking Cessation Group, São Paulo/SP, Brazil; V Philipps University, Department of Pulmonology, Marburg, Germany

**Keywords:** Smoking Cessation, Inflammatory Biomarkers, Respiratory Mucus, Exhaled Breath Condensate, Nasal Lavage

## Abstract

**OBJECTIVE:**

The aim of the present study was to assess nasal mucociliary clearance, mucus properties and inflammation in smokers and subjects enrolled in a Smoking Cessation Program (referred to as quitters).

**METHOD:**

A total of 33 subjects with a median (IQR) smoking history of 34 (20-58) pack years were examined for nasal mucociliary clearance using a saccharine transit test, mucus properties using contact angle and sneeze clearability tests, and quantification of inflammatory and epithelial cells, IL-6 and IL-8 concentrations in nasal lavage fluid. Twenty quitters (mean age: 51 years, 9 male) were assessed at baseline, 1 month, 3 months and 12 months after smoking cessation, and 13 smokers (mean age: 52 years, 6 male) were assessed at baseline and after 12 months. Clinicaltrials.gov: NCT02136550.

**RESULTS:**

Smokers and quitters showed similar demographic characteristics and morbidities. At baseline, all subjects showed impaired nasal mucociliary clearance (mean 17.6 min), although 63% and 85% of the quitters demonstrated significant nasal mucociliary clearance improvement at 1 month and 12 months, respectively. At 12 months, quitters also showed mucus sneeze clearability improvement (∼26%), an increased number of macrophages (2-fold) and no changes in mucus contact angle or cytokine concentrations.

**CONCLUSION:**

This study showed that smoking cessation induced early improvements in nasal mucociliary clearance independent of mucus properties and inflammation. Changes in mucus properties were observed after only 12 months of smoking cessation.

## INTRODUCTION

Tobacco smoking is the most relevant, evitable global risk factor for death. Long-term cigarette smoking is associated with numerous structural and functional alterations in the respiratory system. In the nose and upper airways, long-term cigarette smoking affects cilia structure and function 1-3, which can lead to altered mucociliary clearance (MCC) 4-8. MCC is a primary defense mechanism that protects the human airways and lungs against the harmful effects of inhaled particles. Thus, defects in these defense mechanisms contribute to inflammation and obstruction of the small airways 9 and increased susceptibility to respiratory infections 10,11, lung injury, tissue repair problems, chronic dysfunction and progression of respiratory diseases 12,13.

Smoking cessation is an important part of a comprehensive approach to tobacco control 14 and is the best strategy to avoid respiratory risks and complications, to improve chronic obstructive pulmonary disease (COPD) 15 and to reduce cardiovascular risk and mortality 16-18. A few studies have focused on nasal MCC and inflammation and reported improvements after smoking cessation, although mucus properties have not been investigated. The aim of this study was to assess nasal MCC, mucus properties and inflammation at 1, 3 and 12 months after smoking cessation.

## MATERIAL AND METHODS

### Study population

This study protocol was approved by the Ethical Committee of São Paulo University, São Paulo, Brazil (CEP-FMUSP 235/14) and was conducted in accordance with the Helsinki Declaration (1983). This study is registered at Clinicaltrials.gov (NCT02136550). We recruited subjects (aged above 30 years) from the Ambulatório de Pneumologia, Hospital das Clínicas da Faculdade de Medicina da Universidade de São Paulo, admitted to the Smoking Cessation Program (referred to as quitters). We also recruited smokers from the Faculdade de Medicina da Universidade de São Paulo. The exclusion criteria included the inability to taste saccharine, previous nasal surgery or trauma and infection in the upper and/or lower airways in the 30 days before clinical examination. All participants provided written informed consent.

### Study design

**Figure 1:**
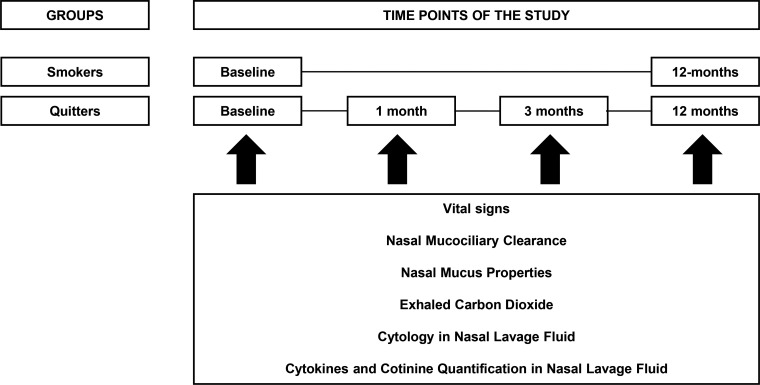
Longitudinal study design.

The subjects were assessed at the University Laboratory of Pulmonary Defense from 1 P.M. until 6 P.M. Smokers and quitters were assessed at baseline and after 12 months. Quitters were also assessed after 1 and 3 months (Figure 1). For all assessments, subjects were asked to avoid coffee, tea, alcohol and green vegetables at least 12 h prior to analysis. We also asked smokers to refrain from smoking cigarettes at least 2 h prior to measurements. Subjects underwent a physical examination with measurements of arterial blood pressure (mmHg), heart rate (bpm) and pulse oximetry (%) after 10 min of resting.

A current smoker was defined as a subject who had smoked ≥100 cigarettes and who currently smoked at least one cigarette per day according to the guidelines of the World Health Organization. Subjects enrolled in our Smoking Cessation Program had received a combination of counselling and medication (nicotine replacement therapy, administration of bupropion and/or nortriptyline) for a 3-month period. All subjects enrolled into this study were investigated for concentrations of exhaled carbon monoxide (CO) and cotinine levels in the nasal lavage fluid (NLF). CO was measured using a Micro CO analyzer (Cardinal Health U.K.232 Ltd., Chatham, UK). Subjects were asked to exhale slowly from their total lung capacity with a constant expiratory flow of 5-6 L.min^-1^ over 10 to 15 sec. Smoking cessation was defined as an exhaled CO <10 ppm and cotinine levels in NLF <10 ng/mL 7.

We also collected demographic data and a clinical history and performed nasal MCC, nasal mucus collection for sneeze clearability and contact angle analysis and NLF collection to measure inflammatory and epithelial cells and cytokine production.

### Nasal mucociliary clearance

The subjects were evaluated in standardized conditions of a quiet room with a 21-23^o^C ambient temperature and 63-71% relative humidity. The subjects remained in a seated position. Briefly, nasal MCC was assessed with the saccharine transit test (STT) 7,19. The STT measures the elapsed time between saccharine deposition 2 cm inside the non-obstructed nostril and the first perception of a sweet taste. We asked subjects to maintain normal ventilation and to avoid deep breaths, coughing, sneezing, sniffing or talking during the test. A normal value for a healthy adult is below 12 min 19.

### Mucus collection and analysis

After the STT, we collected nasal mucus with the aid of a soft brush and stored the sample in the freezer (-80°C) for further analysis of mucus sneeze clearability and the mucus contact angle. To evaluate mucus sneeze clearability *in vitro*, we used a machine that simulates a cough or sneeze, as adapted by King et al. 20. A model acrylic “trachea” cylinder with a cross section of 4 mm and a length of 133 mm was connected to a tank containing pressurized air with a flow of approximately 6 L/s 21. A sample of mucus (25 μl) was deposited in a thin line across the base of the acrylic trachea. Sneeze clearability was measured in millimeters after a single maneuver as the transport distance from the point where the sample was positioned. Wettability refers to the ability to spread over a solid planar surface and is characterized by the contact angle, which is formed between the air-fluid interface and the planar glass. To analyze the contact angle, we used a planar glass pre-treated with a sulphochromic solution and washed with deionized water several times. Five minutes after mucus sample (25 μl) deposition on the planar glass, the measurement of the contact angle was registered using a stereomicroscope (Stemi 2000C, Carl Zeiss, Göttinger, Germany) connected to a camera (Axiocam HSC, Carl Zeiss, Göttinger, Germany) and to a microcomputer with an image program (Interactive AxionVison 4.7, Carl Zeiss, Göttinger, Germany) 7,19.

### Nasal lavage collection

This method has been described previously 7,22. Briefly, subjects were asked to tilt their head back at 30° and to close the nasopharynx with the soft palate. Five milliliters of room temperature isotonic sodium chloride solution (0.9% NaCl) was instilled into each nostril. After 10 sec, the subjects blew their nose forcefully into a sterile plastic container. The average recovery of fluid from the NLF was approximately 70-75%. The NLF was centrifuged (10 min, 300 g, 5°C) and the supernatant was separated from the pellet and divided into five aliquots of 500 μl. The aliquots were coded (for blinding purposes) and stored at –80°C for up to 4 weeks to determine the cytokine levels. The cell pellet was used for total and differential cells counts as previously described 7,22.

### Total and differential cells counts in the NLF

The cell pellet was resuspended in one milliliter of phosphate-buffered saline solution 7,22. Thereafter, 20 µl of the mixed solution was added to a Neubauer chamber, and the cells were counted using a 400x light microscope (Olympus CH2, Olympus America Inc., Palo Alto, USA). For differential cell counts, 100 µl of the mixed solution was centrifuged using a cytospin (96 g, 25°C, 6 min) to obtain two slides for differential cell counts. The slides were stained according to the May–Grunwald–Giemsa method and 100 cells were counted with damaged cells excluded 7,22. The percentages of epithelial cells, neutrophils, eosinophils, lymphocytes and macrophages were calculated as fractions of the total cells. Differential cell counts were performed with the aid of a 1,000x light microscope (Olympus CH2, Olympus America Inc., Palo Alto, USA) by two different observers.

### Measurement of cotinine and cytokines in the NLF

To determine the cotinine levels in the NLF, a high-sensitivity salivary cotinine quantitative enzyme immunoassay (DRG International, Inc., USA) was used according to manufacturer’s instructions. The lower detection limit was 0.1 and the standard curve was fitted between 0 and 50 ng/ml [7)].

The concentrations of interleukin IL-6 and IL-8 in the NLF were determined using high-sensitivity enzyme immunoassays (Quantikine HS, R&D Systems Inc., Minneapolis, USA). The assays were performed as described by the manufacturer. The reported detection limits were 0.039 pg/ml for IL-6 and 0.11 pg/ml for IL-8, with the standard curve fitted between 0 and 10 pg/ml for IL-6 and 0 and 2,000 pg/ml for IL-8.

### Statistical analysis

Data were expressed as the mean ± standard deviation (SD) or median (IQR) when specified. Categorical variables were analyzed by means of the Chi-square test. Analysis of variance (ANOVA) for repeated measures was used to analyze data across the study period with Bonferroni post-hoc tests. Differences were considered statistically significant at a *p*-value <0.05.

## RESULTS

Twenty subjects in each group entered the study. Seven smokers revoked their informed consent and were excluded from the analysis. Twenty quitters and thirteen smokers were followed over 12 months. At baseline, the smokers and quitters showed similar demographic characteristics, frequencies of morbidities and use of medications (hypertensive, beta-blocker and hydroclorotiazide) (Table 1). Furthermore, all current smokers had similar smoking histories of ∼ 42 pack-years. No significant differences were found between smokers and quitters at baseline in systolic blood pressure values (116.1±12.7 and 122.5±11.5 mmHg, respectively), heart rate (82±9 and 81±13 bpm, respectively), and peripheric oxygenation (95±3 and 95±2%, respectively). However, diastolic blood pressure was higher in subjects in the quitter group (∼ 79 mmHg) compared to the smoking group (∼ 73 mmHg, *p*=0.039). These clinical variables did not vary during the study period in either group.

**Figure 2:**
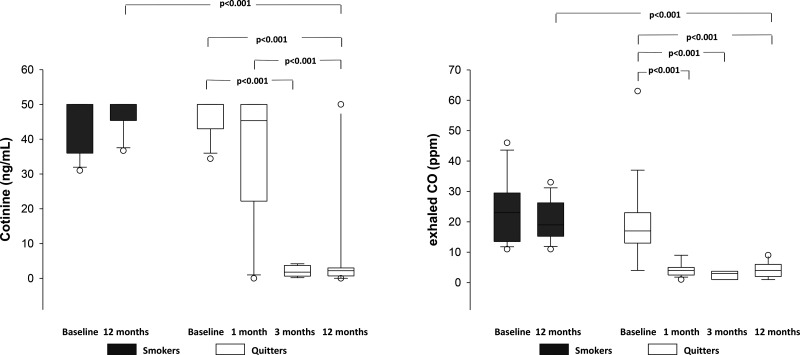
Concentrations of cotinine (ng/mL) and exhaled carbon monoxide (ppm) in smokers and quitters throughout the study.

At baseline, assessments of exhaled CO and cotinine levels in NLF showed similar concentrations in smokers and quitters (Figure 2) One month after quitting with the Smoking Cessation Program, quitters showed a significant decrease in concentrations of exhaled CO that remained low compared with smokers over the observation period of 12 months (Figure 2). Cotinine concentrations in the NLF of quitters decreased after 3 months and remained low after 12 months of smoking cessation.

**Figure 3:**
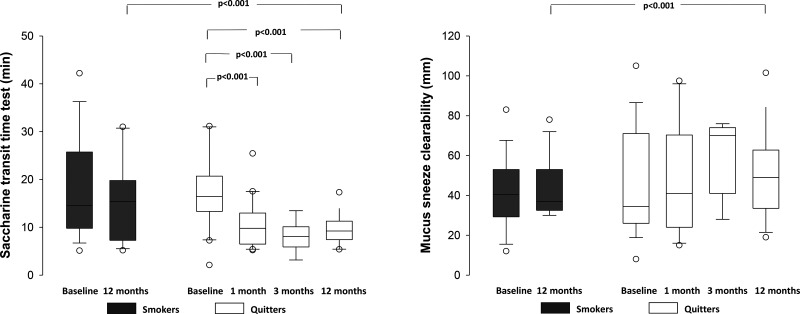
Nasal mucociliary clearance according to the saccharine transit time (min) and mucus clearability by sneeze (mm) in smokers and quitters throughout the study.

At baseline, the STT values were prolonged in both groups (Figure 3). After 12 months, smokers showed a similar pattern of impaired nasal MCC. In contrast, quitters showed significant decreases in STT values from 1 month to 12 months. Mucus clearability by sneeze was similar between groups at baseline. The nasal mucus contact angle was similar between the two groups at baseline and did not change during the study period in smokers (33±7^o^ and 35±10^o^, *p*=0.550) or quitters (36±8^o^ and 32±8^o^, *p*=0.225). However, quitters demonstrated improvements in mucus transportability properties after 12 months (Figure 3).

At baseline, the total numbers of inflammatory and epithelial cells in the NLF were similar between the two groups. However, smokers showed a higher number of macrophages compared with quitters (Table 2). After 12 months, the quitters showed an increase in the number of macrophages in the NLF (*p*<0.001) with no significant differences between groups. The concentrations of IL-6 and IL-8 were similar between groups at baseline and after 12 months.

## DISCUSSION

We conducted this 12-month longitudinal study in long-term smokers to investigate the effects of smoking cessation on nasal MCC, mucus properties and inflammation. Smoking cessation improved nasal MCC after 1 month of recovery, but this improvement was not accompanied by changes in nasal mucus properties or inflammation. Smoking cessation also induced late changes in mucus properties; after 12 months, quitters showed faster mucus clearability by high-flow compared with current smokers.

Tobacco smoking has been associated with increased oxidative stress, airway and lung inflammation 23, impaired anion transport 24 and abnormalities in cilia ultrastructure 1,2,25 and cilia genesis 26. Studies that assessed young smokers 7, adults aged >18 years 27,28 or light smokers (<15 cigarettes/day) 29,30 have shown faster or similar STT results compared with nonsmokers (Table 3 for review). This response may be associated with the preservation of protective mechanisms of airway and lung transport. In young smokers, tobacco smoke induces an early response of ciliary beat frequency acceleration in the nasal ciliated epithelium 31, which in turn results in faster nasal MCC, as previously reported 7. However, the effects of long-term tobacco smoking on MCC remain under debate. Several studies have reported significant STT prolongation in long-term smokers 3-5, and smokers with COPD 35. These impairments may be associated with reductions in ciliary beat frequency 36 or in the number of cilia in the presence of a normal ciliary beat frequency 6. In the current work, 76% of smokers showed prolonged STT at baseline. However, quitting smoking improved nasal MCC by 63% after 1 month and after 3 and 12 months, 85% of subjects reached a normal STT (≤ 12 minutes) with similar values as healthy nonsmokers of the same age 19.

We also focused on changes in mucus properties and inflammation after smoking cessation. Mucus physical properties, ciliary beating frequency and the cilia-mucus interaction are deterministic elements of MCC. We found no changes in mucus surface contact angle during the study in either group, which was consistent with a previous study 7. However, 12 months after quitting smoking, quitters showed increased mucus sneeze transportability. This result is consistent with previous studies showing that mucus viscoelastic properties may be less clearable by cough in smokers compared with nonsmokers 37. We did not find significant changes over 12 months in the number of ciliated cells in the NLF of the two groups of subjects. Some smoking cessation-related mechanistic pathways could explain the early improvement in MCC, such as normalization of cilia structure and genesis 25,26 and ciliary beating function. Mucus clearability by high-flow could be associated with restoration of the ionic transport function and mucus hydration at the basolateral membrane 24. However, ciliary beating frequency, cilia structure and epithelium analysis were beyond the scope of this study.

The present study also found that smokers at baseline showed evidence of nasal inflammation, specifically an increased number of inflammatory cells and cytokine levels in NLF. However, in our previous study with young healthy smokers 7, quitters showed a similar number of macrophages (∼15 cells) compared to nonsmokers (∼14 cells). Thus, we cannot exclude the possibility that quitters in the present study were already reducing their smoking load at the beginning of the study (baseline) or that they could take longer to definitely quit smoking. Additionally, after smoking cessation, there were only small changes in the number of macrophages combined with no changes in pro-inflammatory cytokines (IL-6 and IL-8) in the NLF. However, our results are corroborated by those of others showing maintenance of inflammation in bronchial biopsies of COPD patients 38,39, which can be explained by mechanisms such as airway chronic colonization by viral or bacterial pathogens.

This study has some limitations. First, the sample size of smokers was small and not paired with the quitter group. Indeed, we began with 20 subjects in each group as determined by the sample size calculation. However, seven smokers removed their informed consent after baseline and were excluded from the study. Another limitation was that we may have overestimated the number of subjects who effectively progressed in the smoking cessation program because some subjects may have remained in the quitters group but in fact reinitiated their smoking habit. However, we found it useful to quantify CO in exhaled air and cotinine in the NLF to assess smoking status in the last 2 and 12 hours, respectively, at several time-points of the study and quitters showed a significant decrease in exhaled CO after 1 month of recovery. To highlight differences between the two methods, quitters showed a significant decrease in NLF cotinine levels only after 3 months of recovery. We raise the possibility that this result may be associated with the nicotine replacement therapy delivered during the smoking cessation program. However, we cannot exclude the possibility that subjects may not have adhered to the cessation program after 1 month, taking longer to definitively quit smoking. We did not measure ciliary beat frequency or analyze cilia structure because these methods, such as nasal brushings or biopsies, are difficult and more invasive or traumatic to perform in humans. Another limitation was that counting cells with a two-dimensional method does not take into account the volume of the cell. However, we were able to obtain an inflammatory pattern and epithelial cell profile in both groups. Additionally, we are aware that there are studies showing a reduction in MCC with the use of beta-blockers 40 and furosemide 41 via the blockade of water and ionic transport. However, no subject in the present study used furosemide, and only one smoker (8%) used a beta-blocker, which remained the same throughout the study period.

In conclusion, this study showed early improvement in nasal MCC after smoking cessation. Improvements in mucus properties were observed after 12 months of smoking cessation.

## AUTHOR CONTRIBUTIONS

Utiyama DM, Goto DM and Santos UP performed the literature search, conception and design, acquisition of data, analysis of data and interpretation of data. Yoshida CT performed the literature search, analysis of data and interpretation of data. Nakagawa NK performed the literature search, conception and design, analysis of data and interpretation of data. Saldiva PH contributed to the study conception and design. Utiyama DM, Goto DM, YoshidaCT, Carvalho TS, SantosUP, KoczullaAR, SaldivaPHandNakagawa NK drafted or revised the article and provided final approval of the manuscript.

## Figures and Tables

**Table 1:** Demographic and clinical characteristics of smokers and quitters at baseline of the study are presented as mean ± SD and analyzed by means of T-test (*) or number (proportion) for categorical variables that were analyzed by means of Chi-Square test.

	Smokers n=13	Quitters n=20	*p*-value
**Age**, mean ± SD, years	52±10	51±9	0.860*
**Male**, n (%)	6 (46)	9 (45)	0.948
**BMI**, mean ± SD, kg/m^2^	25.5±4.6	27.8±6.1	0.259*
**Pack-years**, mean ± SD	45±28	40±27	0.450*
**Morbidities**, n (%)			
Hypertension	2 (15)	9 (45)	0.078
Diabetes	0 (0)	1 (5)	0.413
Depression	3 (23)	6 (30)	0.663
Myocardial infarction	1 (8)	1 (5)	0.751
Arrhythmia	1 (8)	0 (0)	0.208
**Medications**, n (%)			
Anti-hypertensive	0 (0)	3 (15)	0.143
Beta-blocker	1 (8)	0 (0)	0.208
Diuretics	1 (8)	2 (10)	0.822

**Table 2:** Total number of inflammatory and epithelial cells and cytokine concentrations (pg/mL) in the nasal lavage fluid of smokers and quitters throughout the study. Data are presented as mean values (SD).

	Smokers n=13		Quitters n=20			
	Baseline	12 months	Baseline	1 month	3 months	12 months
**Total cells x 10^-6^**	74 (85)	97 (60)	86 (200)	36 (26)	85 (87)	58 (37)
Neutrophils	24 (21)	44 (37)	27 (28)	27 (25)	33 (29)	24 (18)
Lymphocytes	9 (7)	7 (7)	10 (10)	11 (9)	9 (6)	7 (5)
Macrophages	33 (14)	27 (20)	15 (11)^ ¥^	19 (14)	17 (11)	44 (15)*
Ciliated cells	34 (15)	21 (17)	21 (25)	37 (23)	22 (22)	21 (5)
Goblet cells	2 (3)	1 (3)	10 (14)	6 (9)	10 (8)	3 (3)
**Cytokines**						
Interleukin-6	2.7 (3.7)	3.9 (4.3)	2.3 (3.2)	3.1 (3.1)	2.0 (2.5)	2.3 (2.0)
Interleukin-8	584.4 (628.9)	770.4 (518.7)	415.7 (589.3)	308.2 (385.4)	352.9 (275.6)	472.4 (398.5)

* *p*<0.001 *vs*. baseline

¥ *p*=0.006 *vs*. other group at the same period of time

**Table 3:** A review of mean (±SD) or median (IQR) values for the saccharine transit time in nonsmokers, passive smokers, current smokers and ex-smokers.

Authors	N Subjects	Age (years)	Smoking habit (pack-year)	STT (min)
Stanley et al. 1986 (3)	Total n=27	33	Nonsmokers	11.1±3.8
			Smokers	20.8±9.3*
Alfaro-Monge & Soda-Merhy 1995 (33)	Total n=100		Nonsmokers	10.3
			Smokers	13.6*
Nakagawa et al. 2005 (5)	Total n=16	32±14	Nonsmokers	10.5
			Smokers	22.0*
Karaman & Tek 2009 (4)	Nonsmokers n=20	18-57	Nonsmokers	12.1±1.9
	Smokers n=20		Smokers > 1	26.4±1.8*
Piotrowska et al. 2010 (32)	Nonsmokers n=21	59 ± 9	Nonsmokers	9.9±0.5
	COPD n=42	50-84	COPD Smokers: 35.8±13.7	16.3±1.6*
			COPD Ex-smokers	11.2±0.5
Ramos et al. 2011 (34)	Nonsmokers n=33	52±14	Nonsmokers	8 (7-13)
	Smokers n=33	49±12	Smokers: 44±25	13 (8-25)*
Ito et al. 2015 (35)	Nonsmokers n=26	60±11	Nonsmokers	8 (6-16)
	Smokers n=27	62±8	Smokers: 38.8±27.6	15.9 (10-27)*
	COPD Ex-smokers n=23	58±8	COPD Ex-smokers: 31.5±24.8	9.7 (6-12)
	COPD Smokers n=17	61±6	COPD Smokers: 39.9±21.4	16.5 (11-28)*
Littlejohn et al. 1992 (27)	Total n=10	> 18	Smokers	11.7±3.3
Mahakit & Pumhirun, 1995 (28)	Total n=40		Nonsmokers	12.0
			Smokers	12.4±3.0
Proença et al. 2012 (30)	Nonsmokers n=30	49 (44-5)	Nonsmokers	8 (8-11)
	Smokers n=52	50 (43-50)	Smokers: 62 (36-78)	10 (10-13)
	Light n=17	51 (41-54)	Light: 23 (23-36)	9 (7-11)
	Moderate n=22	47 (38-49)	Moderate: 15 (13-23)	13 (11-17)
	Heavy n=13	50 (41-57)	Heavy: 25 (19-30)	13 (10-21)
Habesoglu et al. 2012 (29)	Nonsmokers n=15	28±11	Nonsmokers	6.4±1.6
	Passive smokers n=15	29±12	Passive smokers:	12.6±4.7*
			< 10 cigarettes/day	9.3±4.7
			>10 cigarettes/day	15.5±2.0
	Active smokers n=17	28±11	Active smokers	23.6±12.4*
			< 10 cigarettes/day	11.0±3.5
			10-20 cigarettes/day	24.3±3.0
			>20 cigarettes/day	35.0±14.9
Nicola et al. 2014 (7)	Nonsmokers n=32	21±4		7.7±4.1
	Smokers n=40	19±1	Healthy smokers: < 2,5	5.7±3.4*
		24±5	Healthy smokers: > 2,5	5.9±2.9*
Pagliuca et al. 2015 (8)	Nonsmokers n=30	53±6		11.7
	Ex-smokers n=30	51±7	Ex-smokers: 25±8 cigarettes/day	11.8
	Smokers n=3	52±6	Smokers: 25±6 cigarettes/day	15.6
Utiyama et al. 2016	Ex-smokers n-=20	51±9	Ex-smokers: 40±27	8.2±3.1
	Smokers n=13	52±10	Smokers: 45±28	17.9±10.1*

* *p*<0.05 *vs*. nonsmokers
